# Diel and Circadian Patterns of Locomotor Activity in the Adults of Diamondback Moth (*Plutella xylostella*)

**DOI:** 10.3390/insects12080727

**Published:** 2021-08-14

**Authors:** Danfeng Wang, Guang Yang, Wenfeng Chen

**Affiliations:** 1 State Key Laboratory of Ecological Pest Control for Fujian and Taiwan Crops, Institute of Applied Ecology, Fujian Agriculture and Forestry University, Fuzhou 350002, China; adan10@foxmail.com; 2 Joint International Research Laboratory of Ecological Pest Control, Ministry of Education, College of Plant Protection, Fujian Agriculture and Forestry University, Fuzhou 350002, China; 3 Key Laboratory of Integrated Pest Management for Fujian-Taiwan Crops, Ministry of Agriculture and Rural Affairs, College of Plant Protection, Fujian Agriculture and Forestry University, Fuzhou 350002, China; 4 Ministerial and Provincial Joint Innovation Centre for Safety Production of Cross-Strait Crops, College of Plant Protection, Fujian Agriculture and Forestry University, Fuzhou 350002, China; 5 Institute of Life Sciences, College of Biological Science and Engineering, Fuzhou University, Fuzhou 350108, China

**Keywords:** *Plutella xylostella*, locomotor activity, circadian rhythm

## Abstract

**Simple Summary:**

*Plutella xylostell* is a worldwide migratory insect pest that mainly damages cruciferous vegetables. In this study, we established a system for measuring the diel locomotor activities and used it to evaluate the locomotor circadian patterns of *P. xylostella*. We tested the locomotor activities of *P. xylostella* adults under several laboratory settings. We found that both the males and females showed nocturnal activity under a light:dark (LD) cycle, with activity peaking very early after lights off and quickly declining after lights on. Both males and females had high locomotor activity levels in constant darkness (DD) but weak in a constant light condition (LL). In addition, circadian patterns analysis showed that males exhibit much better rhythmic characteristics than females, especially in low temperature conditions. Overall, our proposed system for studying the locomotor activities in *P. xylostella* is reliable, which will help us to have a better understanding of the diel activity of *P. xylostella* and may finally be helpful in the development of an effective pest management strategy.

**Abstract:**

The Diamondback Moth (*Plutella xylostella*) is a highly destructive lepidopteran pest of cruciferous crops. However, there still is relatively little known about the locomotor activities of diel and the circadian patterns in *P. xylostella*. Here, we present an analysis of the diel locomotion of *P. xylostella* under several laboratory settings. We established a system for measuring the individual locomotor activities of *P. xylostella* and found that both males and females showed a nocturnal pattern of activity under 26 or 20 °C LD conditions, with activity peaking immediately after lights off and quickly declining after lights on. In addition, we showed that it is difficult to assess the free-running circadian rhythms of *P. xylostella* under 26 °C DD conditions. However, we found that males showed a higher power, rhythm index (*RI*) and rhythmic ratio than females under 20 °C DD conditions, which indicated that males in low-temperature conditions are much more suitable to study the free-running circadian rhythms of *P. xylostella*. The findings of this study will help us to have a better understanding of the diel activity of *P. xylostella* and may provide a foundation for the development of an effective pest management strategy.

## 1. Introduction

Circadian rhythms are found in most species as the earth rotates around its axis every twenty-four hours. The circadian clock controls daily rhythms in animal physiology and metabolism, thus playing important roles in organismal health and fitness. Insect behaviors, including locomotion, courtship, mating, sperm release, etc., are all affected by the circadian rhythm system [1,2]. Understanding the circadian rhythm of insects, especially lepidopteran pests, has an important reference value for formulating effective pest management strategies [3].

The circadian rhythms control various physiological activities of lepidopteran insects. The circadian rhythms of adult lepidopteran insects have specific characteristics, in which the butterfly species are active during the day, and many moths are active at night [4]. The male gypsy moth (*Lymantria dispar*) exhibits a bimodal rhythm of locomotion and pheromone response, with one peak during the day and one peak at night [5]. This periodic change is affected by the endogenous circadian oscillator. Studies in *Antherea pernyi, Hyalophora cecropia* and *Samia cynthia* have shown that the biological clock controls the rhythm of emergence and migration [1]. The previous studies in *Pectinophora gossypiella* showed that the circadian clock controls the hatching time of eggs [6]. Egg hatching, adult emergence and adult activity showed prominent circadian rhythms in the Mediterranean meal moth (*Ephestia kuehniella*) [7]. In moths, pheromone-mediated reproductive behaviors at specific times have also been extensively studied. Female moths produce and release pheromones at specific times of the day, which is synchronized with the rhythm of male moths; those are sensitive to pheromones [8]. The phototransduction and circadian rhythm synchronization play a key role in the signal transduction mechanism of lepidopteran treetop disease caused by baculovirus [9].

The study in *Drosophila melanogaster* has laid an important foundation to understand the phenomenon of circadian rhythm and related mechanisms [2,10]. In *Drosophila*, the circadian rhythm of locomotor activity is usually measured by using the *Drosophila* Activity Monitoring (DAM) system (TriKinetics; Waltham, MA) [11]. Under light–dark (LD) conditions (usually alternating 12 h light and 12 h dark), flies exhibit a morning activity peak during dawn and an evening activity peak during dusk [2,10]. In constant darkness (DD), the morning peak gradually shrinks; only the evening peak persists and reflecting a circadian clock function with a near-24-hour period [2,10]. At the molecular level, a set of proteins (e.g., PERIOD, CLOCK, CRYPTOCHROME) function as transcription-based feedback loops or post-translational regulation within the clock [2,10]. At the level of the cells, a circadian pacemaker neural network mediates distinct aspects of locomotor behavior [2,10].

However, studies on the biological clock of other insects have shown that there may be some differences from *Drosophila* [12]. The differences in the circadian clock genes in insects stress the complexity in the evolution of clock genes in different insects [13]. *P. xylostella* is a worldwide migratory insect pest that mainly damages cruciferous vegetables and causes economic loss up to USD 4–5 billion annually [14,15]. Here, we explore the locomotor activity rhythms of *P. xylostella* in the laboratory using the DAM system. The main objective of this study is to assess the diel locomotor activities in *P. xylostella* adults. We developed a modified system that can stably monitor the locomotor activities of adult *P. xylostella*. In addition, we discovered robust rhythmic behavior in males but weak rhythmic in females under DD conditions. 

## 2. Materials and Methods

### 2.1. Insect Sample

*P. xylostella* Geneva 88, an insecticide-susceptible strain, was provided by Professor Shelton from Cornell University in 2016 and kept in Fujian Agriculture and Forestry University under 25 ± 1 °C, 70–80% relative humidity and 14L:10D (14 h light:10 h dark) photoperiod. The larvae were reared in a paper cup (10.4 cm × 7.3 cm × 8.5 cm) with an artificial diet. The pupae were transferred to a new paper cup for eclosion. Newly emerged G88 adults were fed with 10% honey water to supplement their nutrition.

### 2.2. Activity Monitor System

A basic system contains the following components: data collection computer, PSIU9 power supply interface unit, activity monitors, tubes and caps and incubator to provide temperature/light stability. Locomotor activity monitors (model number LAM16) and the PSIU9 power supply interface unit were obtained from Trikinetics Inc. (Waltham, MA, USA). Each LAM16 monitor unit has 32 independent channels (16 mm diameter), which were used to load the adults of *P. xylostella* in sampling tubes and to measure locomotor activity using infrared beams and sensors. The sample tubes were made of Polymeric Methyl Methacrylate (PMMA) with a 12-millimeter inside diameter × 100 mm length. Usually, the length of *P. xylostella* adults is about 6 mm. After being occupied by food and stopper, there is about 50 mm length of free space left. After eclosion, the three days old *P. xylostella* were anesthetized with CO_2_ and loaded into the sample tubes at Zeitgeber time 10 (ZT10). Both sexes were kept in the same incubator, and they were able to smell the pheromones of the others. Incubators with a white LED (light-emitting diode) light source were used (light intensity is about 500 lux). LAM16 monitors were connected to the computer with PSIU9 Power Supply Interface Unit. The DAMSystem3 (Waltham, MA, USA) data collection program was set to collect 1-minute bins data from each activity monitor and saved the data in the respective raw monitor file on the hard drive. Then, the FileScan (Waltham, MA, USA) program was used to scan and produce 1-minute bins and 30-minute bins channel files. These files are then ready for analysis.

### 2.3. Locomotor Activity Behaviors Analysis

Rhythmic data can be considered as a wave, and the analysis of locomotor activity data is mainly concerned with a period (the time for a rhythmic event to repeat itself) and amplitude (the robustness of the endogenous clock) [16]. Here, locomotor activities were analyzed using the MATLAB toolboxes developed in Jeffrey Hall’s laboratory [17] and the FaasX developed in François Rouyer’s laboratory [18]. The output of MATLAB toolboxes provides data on the individual’s locomotor activities throughout the experiment in the form of an actogram, an autocorrelation, which calculates the strength of their rhythms using circular statistics [17]. Double-plotted actograms were used to highlight the locomotor activity patterns at different days. Mean activity plots were used to show the activity patterns of 24 h. Autocorrelation plots were used to determine the rhythm index (*RI*), as described previously [17]. Briefly, the oscillation of this autocorrelation function shows periodicity. The asterisk above the third peak of the autocorrelation graph indicates the specific time point used to assess the *RI* [17]. Phase under LD conditions was determined with the FaasX software. Briefly, individuals’ survival at least through the data range requested was used. Data with high frequencies were filtered and the peak was selected as the phase point. Chi square periodogram analysis in FaasX was performed to determine the rhythmicity under DD conditions. Briefly, to justify rhythmicity, Chi-square significance level was set to 0.05, and minimum period peak power and minimum period peak width were set to 20 and 1.5 h, respectively. The parament of power derived from Chi square periodogram analysis reflects the robustness of the endogenous rhythm.

## 3. Results

### 3.1. System for Measuring the Individual Locomotor Activities of P. xylostella

**Figure 1:**
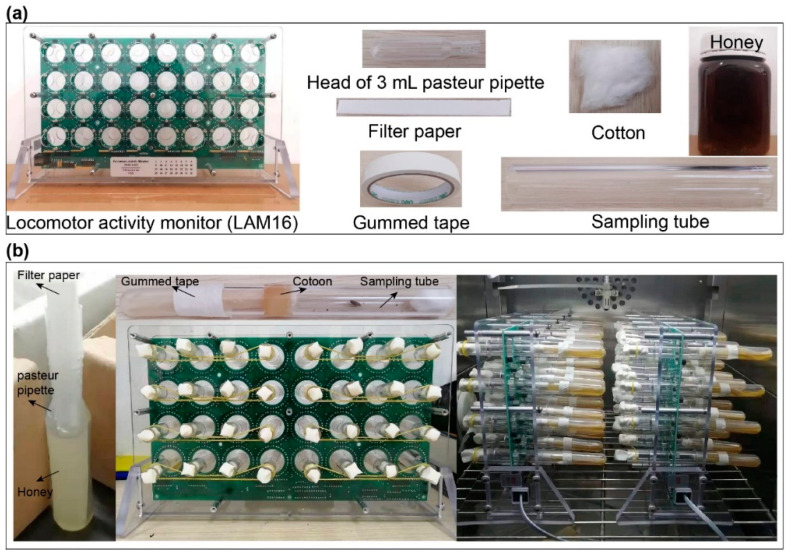
Locomotor activity monitoring system for *P. xylostella*. (**a**) Description of what is contained in the panel (**b**); (**b**) System assembly ensures that every *P. xylostella* individual has a constant supply of honey solution. Fully assembled LAM system, including the food system assembly and the sampling tubes.

The locomotor activity monitors were developed by Trikinetics Inc. (Waltham, MA, USA) and used to quantify animal movement over time [19]. Here, we used model number LAM16 to monitor the circadian rhythm of *P. xylostella*, which has previously been used to measure individual locomotor rhythms in honey bees or mosquitoes [20,21,22] (Figure 1a). In this study, individuals of *P. xylostella* were placed into the tubes with a supply of food, which contains an infrared beam. The computer recorded the locomotor activity as the individuals move back and forth to break the beam. A single activity monitor consists of 32 independent channels, which measure activity using three pairs of infrared emitters and sensors positioned alongside the tubes. The associated electronic components convert analogue information into binary data. One of the main challenges in using the Trikinetics system in *P. xylostella* is the supply of food. We designed a food system that can provide honey food for several days. The food system was built from the head of a 3-milliliter Pasteur pipette, filter paper, cotton, sampling tube, gummed tape and honey (Figure 1a). Firstly, the 2.5-milliliter honey was diluted twice by sterile water, packed into the head of the 3-milliliter Pasteur pipette and 2–3 pieces of suitable size filter paper were put into the diluted honey (Figure 1b). Secondly, the head of the 3-milliliter Pasteur pipette with the honey and filter paper was assembled into one side of the sampling tube and stuffed with a cotton ball to absorb the honey (Figure 1b). Then, the connection between the head of the 3-milliliter Pasteur pipette and the sampling tube was fixed with gummed tape (Figure 1b). Finally, the *P. xylostella* adults were placed in the sampling tube, a stopper was added at the other end of the tubes and then assembled on the LAM16 monitor (Figure 1b).

### 3.2. Locomotor Activities of P. xylostella under Different Temperatures

**Figure 2:**
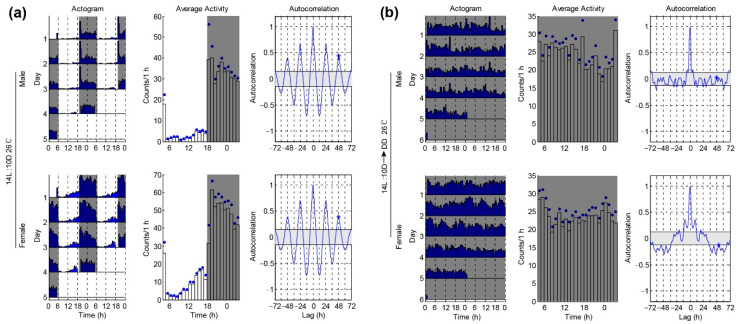
Locomotor activity rhythms of *P. xylostella* male and female at 26 °C. (**a**) Locomotor activity rhythms of *P. xylostella* under the 14L:10D condition. Double-plotted actograms show the locomotor activity patterns of the different days (the left panel). Each row contains two consecutive days of locomotor activities (counts per 30 min), and the last day is repeated to ensure that the next day is always the beginning of the next row. The x-axis shows the day under the light–dark condition. Average activity plots show the activity patterns (the middle panel). The blue dots on the plots are SEM. The gray shadings in the plot denote the light-off condition. Autocorrelation plots are used to determine the rhythm index (*RI*) (right panel). (**b**) Locomotor rhythms of *P. xylostella* under constant darkness.

To test whether the conditions of our activity monitoring system can work well on *P. xylostella*, we started by analyzing the locomotor activities of the male and female moths under standard laboratory conditions. The locomotor activities of the single female and male moth were assessed under 26 °C and 14L:10D conditions for four days, followed by DD for five to six days. Consistent with previous reports that the *P. xylostella* adults are usually active at twilight and throughout the night [23], we found a nocturnal pattern of activity under LD, with peaking immediately after lights off and quickly declining after lights on (Figure 2a). The autocorrelation analysis of the circadian locomotor activities under LD revealed that both the female and male moth had a period of nearly 24 h (male 24.03 ± 0.16 h, female 24.07 ± 0.16 h), and both had an average rhythm index (RI) of about 0.3 (male 0.28 ± 0.02, female 0.26 ± 0.02) (Figure 2a and Table 1). The peak phase or average activity analysis was comparable in the males and females (Table 1 and Table 2). In DD conditions, we found that both the male and female moths had high locomotor activity levels, either in the subjective day or night (Figure 2b). However, the analysis of the locomotor activity rhythms under DD revealed that they were highly arrhythmic (male arrhythmic = 21/29 vs. female arrhythmic = 33/35). Autocorrelation analysis showed that both the female and male moth had very weak oscillation in the period, and the average RIs of the males and females were only 0.15 ± 0.01 and 0.12 ± 0.02, respectively (Figure 2b and Table 1). Although the Chi square periodogram analysis of rhythmic individuals showed that the males have higher power than the females under the DD condition (Table 1), all of these data indicate that it is difficult to assess the free-running circadian rhythms of *P. xylostella* under the 26 °C condition.

**Figure 3:**
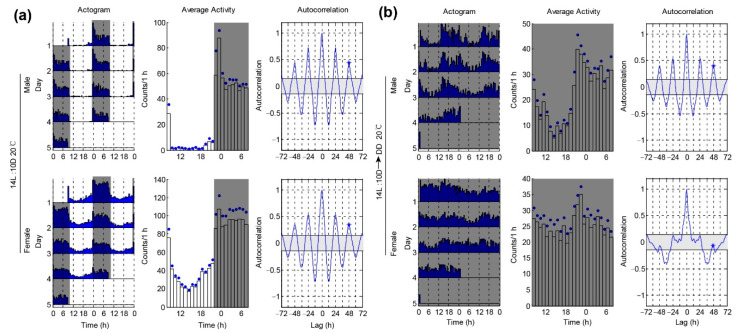
*P. xylostella* males exhibit better rhythm than females under 20 °C. (**a**) Locomotor activity rhythms of *P. xylostella* under 14L: 10D and 20 °C conditions. Double-plotted actograms show the locomotor activity patterns of the different days (left panel). Each row contains two consecutive days of locomotor activities (counts per 30 min), and the last day is repeated to ensure that the next day is always the beginning of the next row. The x-axis shows the time of day under LD conditions. Mean activity plots were used to show the activity patterns (middle panel). The blue dots on the plots are SEM. The gray shadings in the plots denote light-off conditions. Autocorrelation plots are used to determine the rhythm index (*RI*) (right panel). (**b**) Locomotor activity rhythms of the *P. xylostella* under the changing condition from 14L:10D to DD at 20 °C.

We then decided to test whether temperature compensation is involved in affecting the locomotor rhythm under DD. Temperature compensation enables organisms to maintain robust rhythms with a period close to a day over a wide range of physiological temperatures. We set the locomotor activities of single females and males *P. xylostella* under 20 °C and 14L:10D conditions. Similar to the results of 26 °C, we still found that both the male and female moths showed a nocturnal pattern of locomotion under LD and had similar phases (male, 2.0 ± 0.6 h; female, 2.4 ± 1.1 h) (Figure 3a and Table 1). Furthermore, we found that the average activity levels of the males and females were similar (Figure 3b and Table 2). The arrhythmic ratio of the females was significantly higher than that of the males under 20 °C and DD conditions (male arrhythmic = 20/38 vs. female arrhythmic = 36/41) (Table 1). The average power and RI of the males under DD were also significantly higher than the females (Figure 3b and Table 1). These results indicated that the males had a better rhythm than the females under a low temperature. This finding is surprising because the ability to maintain a fixed endogenous period throughout a range of physiological temperatures (temperature compensation) is a defining feature of circadian rhythms. Subsequently, we carried out the experiments under 20 °C.

### 3.3. Locomotor Activities of P. xylostella under a Winter-like Short Day

**Figure 4:**
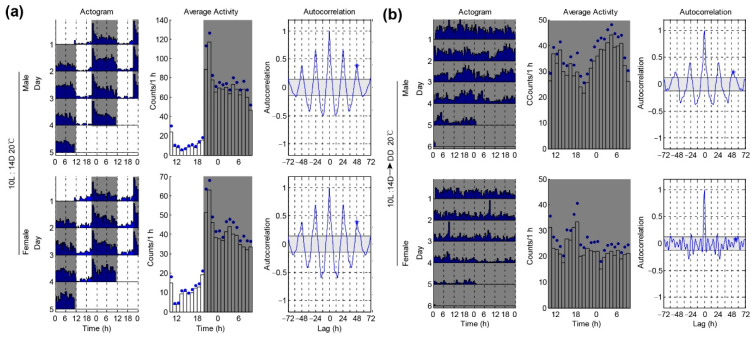
*P. xylostella* males show better rhythm than females under a winter-like short day at 20 °C. (**a**) Locomotor rhythm of *P. xylostella* under 10L:14D and 20 °C conditions. Double-plotted actograms show the locomotor activity patterns of the different days (left panel). Each row contains two consecutive days of locomotor activities (counts per 30 min), and the last day is repeated to ensure that the next day is always the beginning of the next row. The x-axis shows the time of day under LD conditions. Mean activity plots are used to show the activity patterns (middle panel). The blue dots on the plots are SEM. The gray shadings in the plots denote light-off conditions. Autocorrelation plots are used to determine the rhythm index (*RI*) (right panel). (**b**) Locomotor activity rhythms of *P. xylostella* under the changing condition from 10L:14D condition to DD at 20 °C.

Light is the dominant environmental cue that provides temporal information to circadian pacemakers. Here, we wondered whether the photoperiod affects the locomotor rhythm of *P. xylostella*. In the former 14L:10D condition, we provided a summer-like long day. Next, we set a winter-like short day condition with 10L:14D. We found that both the male and female moths still showed nocturnal patterns under 10L:14D and had comparable peak phases (Figure 4a and Table 1). Similar to the results under 14L:10D, the females still showed a higher arrhythmic ratio than the males when transferred from 10L:14D to DD (male arrhythmic = 10/21 vs. female arrhythmic = 20/20) (Figure 4b and Table 1). The average power and RI of the males under DD were also significantly higher than the females (Figure 4b and Table 1). Together, these data show that *P. xylostella* under a winter-like short day had a similar locomotor rhythm as the moths under a summer-like long day (Figure 3 and Figure 4).

### 3.4. Locomotor Activities of P. xylostella under Constant Light Condition

**Figure 5:**
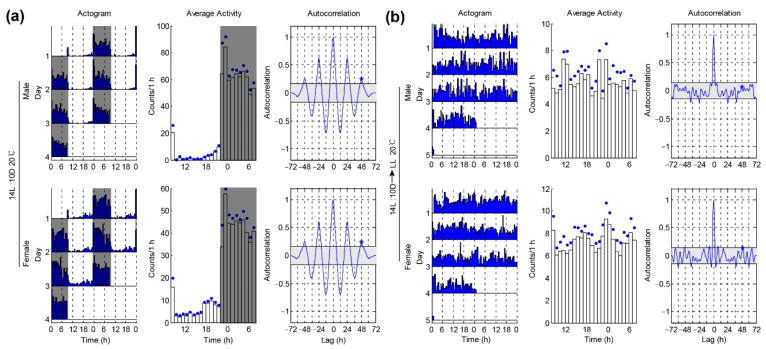
Both *P. xylostella* males and females exhibit arrhythmic when transferred from 14L:10D condition to LL. (**a**) Locomotor rhythm of *P. xylostella* under 14L:10D condition. Double-plotted actograms show the locomotor activity patterns of the different days (left panel). Each row contains two consecutive days of locomotor activities (counts per 30 min), and the last day is repeated to ensure that the next day is always the beginning of the next row. The x-axis shows the time of day under light-dark conditions. Average activity plots used to show the activity patterns (middle panel). The blue dots on the plots are SEM. The gray shadings in the plots denote light-off conditions. Autocorrelation plots are used to determine the rhythm index (*RI*) (right panel). (**b**) Locomotor rhythms of *P. xylostella* under the changing condition from 14L:10D condition to constant light.

In *Drosophila melanogaster*, constant light (LL) conditions stop the molecular cycling and cause wild-type flies to become arrhythmic [24]. To test whether the *P. xylostella* has a rhythm activity pattern under the LL condition, we kept the *P. xylostella* individuals under LD condition for four days and then transferred them to LL. Both the male and female moths showed the normal nocturnal activity pattern during LD (Figure 5a). However, in contrast to the males with normal rhythm under DD conditions, we found that both the males and females showed arrhythmic activity in LL with only about 6–8 counts of activity events every 1 h (Figure 5b and Table 1 and Table 2). These data indicate that light is an important factor in inhibiting the locomotor activities of *P. xylostella*.

## 4. Discussion

Most insects show unimodal activity patterns [25]. However, small insect species with crepuscular habits are bimodal. The most important species with these aspects are *Drosophila* and mosquitoes. Many *Drosophila* species show a bimodal activity rhythm with peaks close to dawn and dusk, and a suppression of activity around noon [2,10]. *Drosophila* gradually increases their locomotor activity in advance of both dark-to-light and light-to-dark transitions, a phenomenon termed anticipation [2,10]. Interestingly, our data show that *P. xylostella* also appears to increase their activity gradually before switching from light to dark (see the average activity plots under LD conditions). Most of the moths show unimodal activity patterns may peak at different times. Here, we found the rhythm of the male and female locomotor activity reaching the peak at dusk in *P. xylostella*, whether under the long-day (14L:10D) or the short-day (10L:14D) conditions. Similar to our results, circadian locomotor activity in the adult fall armyworm, *Spodoptera frugiperda*, shows activity during darkness, with activity immediately after lights-off [26]. Both the Indian meal moth, *Plodia interpunctella* and the Mediterranean flour moth, *Ephestia kuehniella* show daily rhythms in calling and locomotor behaviors [27]. The rhythm of male locomotor activity reaches the peak at dusk in *P. interpunctella* and dawn *in E. kuehniella* [27]. However, under long-day conditions (17L:7D), both *P. interpunctella* and *E. kuehniella* tend to display bimodal activity patterns [28]. Whether *P. xylostella* will also show bimodal pattern under longer daytime is worthy of being studied further. Interestingly, the rhythms of calling behavior turn arrhythmic in *P. interpunctella* females in DD, whereas *E. kuehniella* females showed a persistent rhythm in DD, suggesting a different circadian clock regulation mechanism of moths [27]. On the other hand, a lot of the general activity level of *P. xylostella* can be explained by just the absence/presence of light. The activity, at least in the females, seems to be a direct light response (inhibition of locomotor activity) rather than a circadian-driven behavior. In the future, we may be able to study this question by making mutants of different clock genes.

It has been reported that different behaviors in insects are affected by temperature change. The circadian rhythm of locomotor activity under DD has been observed in many insect species and is now regarded as a property of the endogenous system [29]. However, we found that both *P. xylostella* males and females showed highly arrhythmic free-running behavior under 26 °C. In contrast, males show better locomotor rhythm under 20 °C. Circadian clocks are synchronized with both light:dark cycles and temperature fluctuations. Temperature changes may affect the clock mechanism by changing the component process, as seen in *Drosophila* and other insects. The *Drosophila* clock genes can be entrained by temperature cycles [28]. Antennal temperature signals regulated by the TRPA channel PYREXIA synchronize clock neurons in the brain to regulate the *Drosophila* circadian clock protein PERIOD [30]. Similar to locomotor rhythm, it has been reported that the adult emergence rhythm can also be affected by different temperature cycles [31]. The peaks of emergence in *P. xylostella* were delayed with the increase in temperature [32]. Temperature affects the sexual activity of moths generally, such that at lower temperatures females call earlier, and that a decrease in temperature may lift the inhibition of calling instead of inducing calling [8]. Temperature can also play a critical role in the circadian system to control sperm release in moths [33]. Measuring the locomotor rhythm of the honey bee at different temperatures reveals that testing the endogenous rhythm at 35 °C results in periods closer to 24 h compared with 25 °C [20]. These findings suggest that the degree of tuning of circadian temperature compensation may vary among different species.

According to ‘Aschoff’s rule’, τ lengthens with an increase in light intensity, or on transfer from DD to LL, for dark-active animals (i.e., τDD < τLL, nocturnal), but shortens for light-active animals (i.e., τDD > τLL, diurnal) [34]. However, the general applicability of this ‘rule’ in insects is in doubt. In some nocturnal species, the results for a transfer from DD to LL, and vice versa, are generally in agreement with ‘Aschoff s rule’. Locomotor and flight activity rhythms frequently become arrhythmic in LL at higher light intensities. Here, we also found that *P. xylostella* became arrhythmic in LL. However, whether light intensity affects the rhythm of *P. xylostella* under LL needs to be studied further.

Understanding the biological clocks of insects could help us to develop more effective pest control strategies. The circadian rhythms of certain insects make them more susceptible to insecticides at certain times of the day than at other times [35], because the susceptibility to insecticide can be influenced by the rhythmic activity of xenobiotic metabolizing (XM) enzymes. For example, the enzymatic assay of glutathione S-transferase (GST), esterase and P450 in *Cimex lectularius* reveal significant time-of-day specific changes that have similar peak phases with the highest activity consistently recorded during the late photophase at ZT9 in LD [36]. The XM gene expression assays also reveal significant time-of-day differences in mRNA expression patterns in both LD and DD. Here, for the first time, we systematically studied the locomotor circadian rhythm of the *P. xylostella*. We believe that our results will be helpful in the future to clarify the molecular mechanism of the circadian rhythm of the *P. xylostella* and finally be helpful in the development of an effective pest management strategy.

## 5. Conclusions

A system for measuring the individual locomotor rhythm of *P. xylostella* is established. Both the males and females show a nocturnal pattern of activity, with activity peaking very early after lights off and quickly declining after lights on. It is difficult to assess the free-running circadian rhythms of *P. xylostella* under 26 °C DD conditions. Males in low-temperature conditions are much more suitable to study the free-running circadian rhythms of *P. xylostella*.

## Figures and Tables

**Table 1:** Locomotor activity circadian rhythms of *P. xylostella* under different light conditions.

Condition	Phase (95% Confidence Limit)		Power	Average Rhythm Index (RI)	Phase (95% Confidence Limit)		Power	Average Rhythm Index (RI)
	Male				Female			
	LD (4 days)							
26 °C 14L:10D	1.4 ± 1.1		54.2 ± 3.07 ^n.s.^	0.28 ± 0.02 ^n.s.^	2.8 ± 0.8		50.4 ± 3.07	0.26 ± 0.02
20 °C 14L:10D	2.0 ± 0.6		70.9 ± 3.98 **	0.31 ± 0.02 **	2.4 ± 1.1		54.5 ± 3.36	0.23 ± 0.02
20 °C 10L:14D	22.5 ± 0.9		55.9 ± 2.87 *	0.29 ± 0.02 **	22.0 ± 0.3		50.4 ± 3.81	0.16 ± 0.04
**Condition**	**Arrhythmic Ratio**	**Period**	**Power**	**Average Rhythm Index (RI)**	**Arrhythmic Ratio**	**Period**	**Power**	**Average Rhythm Index (RI)**
	LD-DD or LD-LL (5–6 days)							
26 °C 14L:10D-DD	21/29 *	24.3 ± 2.85	41.3 ± 6.07 *	0.15 ± 0.01 ^n.s.^	33/35	25.8 ± 1.25	25.7 ± 2.58	0.12 ± 0.02
20 °C 14L:10D-DD	20/38 ***	24.0 ± 0.31	46.4 ± 3.80 **	0.19 ± 0.01 **	36/41	27.0 ± 1.81	37.6 ± 3.47	0.14 ± 0.02
20 °C 10L:14D-DD	10/21 ***	23.8 ± 0.28	36.6 ± 6.35	0.16 ± 0.02 **	20/20	-	-	0.10 ± 0.03
20 °C 14L:10D-LL	20/20	-	-	-	16/16	-	-	-

Note: Period, power and arrhythmic ratio were calculated using Chi-square periodogram analysis. Individual survival at least through the data range requested were used to perform analysis, and only the rhythmic individuals were used to calculate period and power. Rhythm index was calculated using autocorrelation analysis (all survival individuals were used). Comparisons were made between males and females with a two-tailed, unpaired Student’s *t*-test. * *p* < 0.05, ** *p* < 0.01, *** *p* < 0.001, ^n.s.^ no significance.

**Table 2:** Daily activity levels of *P. xylostella* under different light conditions.

Condition	Number	Activity: Average/30 min	Number	Activity: Average/30 min
	Male		Female	
	LD (4 days)			
26 °C 14L:10D	42	31.5 ± 4.05	46	30.4 ± 2.94
20 °C 14L:10D	40	35.8 ± 3.22	42	31.7 ± 3.62
20 °C 10L:14D	30	46.5 ± 4.55	28	26.7 ± 2.81
**Condition**	**Number**	**Activity: Average/30 min**	**Number**	**Activity: Average/30 min**
	DD (4 days) or LL (4 days)			
26 °C 14L:10D-DD	29	23.9 ± 3.11	35	26.0 ± 3.00
20 °C 14L:10D-DD	38	20.3 ± 3.07	41	15.6 ± 2.31
20 °C 10L:14D-DD	21	24.7 ± 2.85	20	19.3 ± 2.53
20 °C 14L:10D-LL	20	6.1 ± 1.44	16	8.2 ± 1.53

## Data Availability

All data is available in this paper.
